# Worsening of asthma control after recovery from mild to moderate COVID-19 in patients from Hong Kong

**DOI:** 10.1186/s12931-023-02363-z

**Published:** 2023-02-14

**Authors:** Wang Chun Kwok, Terence Chi Chun Tam, David Chi Leung Lam, Jackson Ka Chun Leung, King Pui Florence Chan, Shung Kay Samuel Chan, Ka Yan Chiang, Mary Sau Man Ip, James Chung Man Ho

**Affiliations:** Department of Medicine, The University of Hong Kong, Queen Mary Hospital, 4/F, Professorial Block, 102 Pokfulam Road, Pokfulam, Hong Kong, Special Administrative Region China

**Keywords:** COVID-19, Asthma, Asthma control, Asthma exacerbation

## Abstract

**Background:**

While there are postulations that asthma is potentially associated with severe coronavirus disease 2019 (COVID-19), there has been conflicting results from studies on the impact mild-to-moderate COVID-19 on asthma control after recovery.

**Methods:**

A case control study on the association between mild-to-moderate COVID-19 and asthma control post infection was conducted. The primary outcome was a reduction in Asthma Control Test (ACT) score by ≥ 3 points post-COVID infection. The secondary outcomes included the change in ACT score, the proportion of patient with ACT score who dropped to ≤ 15 on enrolment visit and the need for escalation of asthma maintenance therapy.

**Results:**

Out of the total of 221 adult patients with asthma recruited, 111 had mild-to-moderate COVID-19 within 30 to 270 days prior to study enrolment. The adjusted odds ratio (aOR) for a reduction in ACT score by ≥ 3 points after COVID-19 was 3.105 (95% CI = 1.385–6.959, p = 0.006). The odds of escalation of asthma maintenance therapy by at least 1 Global Initiative for Asthma (GINA) step was 4.733 (95% CI = 1.151–19.467, p = 0.031) and asthma patient are more likely to become uncontrolled after COVID-19 [aOR = 5.509 (95% CI = 1.061–28.600, p = 0.042)].

**Conclusion:**

Mild-to-moderate COVID-19 among asthma patients, upon recovery, was associated with worsening of asthma symptom, lower ACT score, a higher need for escalation of asthma maintenance therapy and more uncontrolled asthma.

## Background

Asthma is one of the commonest respiratory diseases around the world and viral respiratory infection is one of the most frequent triggers for asthma exacerbation [1]. To assess asthma control, asthma control test (ACT) is frequently being used as it is a validated simple questionnaire that can be self-completed for disease monitoring. It consists of 5 questions on various domains of asthma control, with a higher total score (range:5–25) indicating better asthma control [2]. The evidence on the association between asthma and coronavirus disease 2019 (COVID-19) is controversial [3–8]. While pediatric-focused study suggested that asthma control was unaffected by COVID-19, there are limited data in the adult population [9]. An adult study conducted in the United Kingdom suggested that persisting symptoms and increased inhaler use are common in people with asthma following COVID-19, but since the study was in a form of online survey it could suffer heavily from both reporter and non-responder bias [10]. In addition, the conclusions reached from these studies, which were conducted on or prior to 2021, may not be entirely applicable to the current time as the prevalent COVID-19 variant had now changed and there was no effective and widespread use of oral antivirals back then [11]. A more contemporary study published in 2022 suggested that there was significant more patients who have worsening of asthma control after they recovered from COVID-19; however, this study did not stratify the degree of exacerbation nor was ACT changes measured prior and after COVID-19 infection [12].

We hereby conducted this cross-sectional case–control study to assess the association between mild-to-moderate COVID-19 infection, and asthma control upon infection recovery with detailed and objective assessment of asthma control over various clinical parameters.

## Methods

### Study design and data sources

There are approximately 950 patients with asthma that is regularly followed up in asthma clinic in Queen Mary Hospital, and potentially eligible subjects were identified from this clinic during the enrolment period from 24th May 2022 to 1st November 2022. Eligible subjects were adults aged ≥ 18 years with asthma. The subjects who had scheduled follow-up in asthma clinic were recruited. Patients who had ad hoc or emergency visits in asthma clinic after COVID-19 were excluded to avoid selection bias. Those who had mild to moderate COVID-19 30 to 270 days before the date of assessment were classified into the COVID-19 group, while equal number of controls that did not have COVID-19 that were matched based on age, gender, smoking status, asthma severity and lung function were included and classified in non-COVID-19 group. Mild disease is defined as patients who have any of the various signs and symptoms of COVID-19 (e.g., fever, cough, sore throat, malaise, headache, muscle pain, nausea, vomiting, diarrhea, loss of taste and smell) but who do not have shortness of breath, dyspnea, or abnormal chest imaging. Moderate illness is defined as patients who show evidence of lower respiratory disease during clinical assessment or imaging and who have an oxygen saturation measured by pulse oximetry (SpO_2_) ≥ 94% on room air at sea level [13]. In Hong Kong, for non-hospitalized patients with COVID-19 need to have at least 7 days home quarantine if they completed COVID-19 vaccine. The quarantine period is at least 14 days among those who have not completed COVID-19 vaccination. They are not allowed to leave their residence until the quarantine order is off. This is reinforced by the use of Leave Home Safe apps which will incorporate both the vaccination record and the infection record. As such, they will not be able to attend the asthma clinic in Queen Mary Hospital during the quarantine period. All patients who were included in this study were all from the asthma clinic, which the patients attending were all out of the acute infection who are allowed to go to hospital by law. Patient with asthma-COPD overlap, history of severe COVID-19 that required oxygen therapy, mechanical ventilation or intensive care unit admission were excluded. The diagnosis of COVID-19 was confirmed by laboratory-confirmed positive reverse transcription–polymerase chain reaction (RT-PCR) test, or positive rapid antigen test (RAT), as documented on the designated COVID-19 data platform on Clinical Management System (CMS) of Hong Kong Hospital Authority. Patients’ records were accessed through the electronic patient record (ePR) of the Hong Kong Hospital Authority, which consisted of the records of all patients with out-patient clinic attendances and hospital admissions. The information available included patient demographics, clinical notes, investigation results and treatment records. Demographic data (age, gender, smoking status) and clinical data / investigations (ACT score, asthma medication, comorbidities, spirometry results, date of COVID-19, hospitalization and complications from COVID-19, date and dose of COVID-19 vaccination, type of COVID-19 vaccines) were identified from clinical records. The past ACT score, asthma medication, comorbidities, spirometry results and COVID-19 vaccination details were collected from a retrospective database. ACT scores range from 5 (poor control of asthma) to 25 (complete control of asthma), and the minimally important difference (MID) is 3 points [14]. Asthma is classified as well controlled if ACT score is 20 or above, partially controlled if ACT score is 16 to 19 and uncontrolled if ACT score is 15 or below [15, 16]. Spirometry was performed with CareFusion Vmax® Encore 22 system. Spirometry data was interpreted with the updated spirometric reference values for adult Chinese in Hong Kong [17]. The study was approved by the Institutional Review Board of the University of Hong Kong and Hospital Authority Hong Kong West Cluster (UW 22-110).

### Outcomes

The primary outcome was the deterioration in asthma control defined as a reduction in ACT score by three or more point from “the immediate prior visit” to “enrolment visit”. The secondary outcomes include the magnitude of change in ACT score at enrolment visit, the proportion of patients with ACT score at or below 15 at enrolment visit and the need for escalation of asthma maintenance therapy.

### Statistical analysis

The demographic and clinical data were described in actual frequency, mean ± SD or median (interquartile range). Baseline demographic and clinical data were compared between the patients with or without COVID-19 by Chi-squared test or Fisher’s exact test as appropriate. Continuous variables are expressed as mean ± standard deviation (SD) and compared using the student’s t test. The risks of worsening asthma control between patients with or without COVID-19 will be compared by binary logistic regression. Multiple logistic regression modeling was used to account for potential confounders including age, gender, smoking status, baseline FEV_1_ (% predicted), COVID-19 vaccination status, ACT score at prior visit 12 months before and GINA step of medication at baseline. The statistical significance was determined at the level of p < 0.05. All the statistical analyses will be done using the 26th version of SPSS statistical package.

## Results

A total of 221 adult patients with asthma were recruited over the enrolment period of which 111 (50.0%) can be classified into the COVID-19 group. In those with COVID-19 infection, 5 required hospitalizations, and none had severe COVID-19.

Among the whole cohort of 221 patients included in the analysis, the mean age was 58.0 ± 16.0 years. There were 81 (36.7%) male patients and 179 (81.0%) were never smoker. 5 (2.3%), 22 (10.0%), 78 (35.3%), 74 (33.5%), 42 (19.0) had GINA step 1, 2, 3, 4 and 5 medications at baseline. Co-existing rhinosinusitis and atopic dermatitis was identified in 177 (80.1%) and 86 (38.9%) patients respectively. The mean baseline FEV_1_ was 2.08 ± 0.80 L (88.6 ± 23.3% predicted), with the baseline FEV_1_/FVC ratio was 67.0 ± 14.7%. The mean ACT score was 20.0 ± 4.0 and 19.0 ± 4.9 at the immediate prior visit and enrolment visit, respectively. A total of 53 (24.0%) patients had asthma exacerbation in the 12-months period prior to enrolment visit. The mean baseline eosinophil count was 288 ± 247 cells/µL while the median serum IgE level was 166.5 [Interquartile range = 85.25–529.75]. The mean time from immediate prior visit to enrollment visit was 185 ± 72 days. The mean time of the enrolment visit after the diagnosis of COVID-19 was 47 ± 57 days. One hundred and ninety-eight (89.6%) patients completed at least 2 doses of COVID-19 vaccines. The baseline demographics were listed in Table 1.

**Table 1:** Baseline demographic and clinical characteristics of included patients

	COVID-19 (n = 111)	Non COVID-19 (n = 110)	Whole cohort (n = 221)	p-values^
Age (years), mean ± SD	56.6 ± 16.8	59.4 ± 15.2	58.0 ± 16.0	0.197
Age of asthma onset (years), mean ± SD	27.1 ± 23.3	28.4 ± 22.2	27.8 ± 22.7	0.688
Gender				0.675
Male	42 (38.2%)	39 (35.5%)	81 (36.8%)	
Female	68 (61.8%)	71 (64.5%)	139 (63.2%)	
Smoking status				0.808
Non-smoker	88 (79.3%)	91 (82.7%)	179 (81.0%)	
Active smoker	11 (9.9%)	9 (8.2%)	20 (9.0%)	
Former smoker	12 (10.8%)	10 (9.1%)	22 (10.0%)	
Co-morbidities				
Rhinosinusitis	95 (85.6%)	82 (74.5%)	177 (80.1%)	0.059
Atopic dermatitis	41 (36.9%)	45 (40.9%)	86 (38.9%)	0.545
GINA steps				0.353
1	4 (3.6%)	1 (0.9%)	5 (2.3%)	
2	13 (11.7%)	9 (8.2%)	22 (10.0%)	
3	35 (31.5%)	43 (39.1%)	78 (35.3%)	
4	35 (31.5%)	39 (35.5%)	74 (33.5%)	
5	24 (21.6%)	18 (16.4%)	42 (19.0%)	
Exacerbation required medical attendance in past 12 months before enrolment	28 (25.2%)	25 (22.7%)	53 (24.0%)	0.664
Exacerbation required systemic corticosteroid in past 12 months before enrolment	22 (19.8%)	21 (19.1%)	43 (19.5%)	0.891
Completion of COVID-19 vaccine (More than 2 doses for more than 14 days)	96 (86.5%)	102 (92.7%)	198 (89.6%)	0.129
Baseline FEV_1_ (L), mean ± SD	2.08 ± 0.86	2.07 ± 0.76	2.08 ± 0.80	0.900
Baseline FEV_1_ (% predicted), mean ± SD	87.2 ± 22.7	89.8 ± 23.9	88.6 ± 23.3	0.471
Baseline FVC (L), mean ± SD	3.08 ± 1.00	3.11 ± 0.95	3.10 ± 0.97	0.866
Baseline FVC (% predicted), mean ± SD	103.4 ± 18.9	115.8 ± 96.2	109.9 ± 71.2	0.231
Baseline FEV_1_ to FVC ratio, mean ± SD	67.3 ± 15.4	66.7 ± 14.2	67.0 ± 14.7	0.788
Baseline eosinophil count (x cells/µL), mean ± SD	312 ± 261	264 ± 230	288 ± 247	0.156
Serum IgE level, median [IQR]	176.5 [94.5–731.5]	148.5 [72–433.75]	166.5 [85.25–529.75]	0.488
ACT at immediate prior visit, mean ± SD	20.0 ± 4.5	20.0 ± 3.5	20.0 ± 4.0	0.867
ACT at enrolment visit, mean ± SD	17.5 ± 5.3	20.4 ± 3.9	19.0 ± 4.9	< 0.001*
Asthma control by ACT score at immediate prior visit				0.549
Controlled	67 (60.4%)	64 (58.2%)	131 (59.3%)	
Partially controlled	27 (24.3%)	33 (30.0%)	60 (27.1%)	
Uncontrolled	17 (15.3%)	13 (11.8%)	30 (13.6%)	
Asthma control by ACT score at enrolment visit				0.039*
Controlled	52 (46.8%)	55 (50.0%)	107 (48.4%)	
Partially controlled	37 (33.3%)	46 (41.8%)	83 (37.6%)	
Uncontrolled	22 (19.8%)	9 (8.2%)	31 (14.0%)	
Change in ACT score over 12 months, mean ± SD	− 2.47 ± 4.96	+ 0.40 ± 3.46		< 0.001*
> 3 points decrease in ACT score	42 (37.8%)	12 (12.7%)	54 (24.4%)	< 0.001*
Escalation of asthma maintenance therapy by at least 1 GINA step	17 (15.3%)	5 (4.5%)	22 (10.0%)	0.008*

*SD* standard deviation, *IQR* interquartile range, *µL* microliters, *L* liter, *ACT* asthma control test, *FEV*_*1*_ forced expiratory volume in one second, *FVC* forced vital capacity, *GINA* global Initiative for Asthma

^Between non-COVID-19 and COVID-19 subgroup

*Statistically significant

### A reduction in ACT score by ≥ 3 points

Overall, 56 (25.3%) of the patients had a reduction of ACT by ≥ 3 points at enrolment visits with greater proportion observed in those who had COVID-19 than those who did not (37.8% vs. 12.7%). The odds ratio (OR) by univariate logistic regression was 4.174 (95% confidence interval [CI] = 2.116–8.234, p < 0.001), and was 3.105 (95% CI = 1.385–6.959, p = 0.006) after adjusting for potential confounders including age, gender, smoking status, baseline FEV_1_ (% predicted), COVID-19 vaccination status, ACT score at prior visit 12 months before and GINA step of medication at baseline. The results were summarized in Table 2.

**Table 2:** Odds-ratio (OR) of clinical parameters for worsening of asthma control

	OR	95% C.I	p-value	aOR	95% C.I	p-value
≥ 3 points decrease in ACT score	4.174	2.116–8.234	< 0.001*	3.105	1.385–6.959	0.006*
Escalation of asthma maintenance therapy by at least 1 GINA step	3.798	1.349–10.694	0.012*	4.733	1.151–19.467	0.031*
Asthma uncontrolled	2.774	1.214–6.338	0.016*	5.509	1.061–28.600	0.042*
Asthma from controlled to uncontrolled	8.209	3.018–22.233	< 0.001*	7.532	2.268–25.013	< 0.001*
Medical attendance for asthmatic exacerbation	1.083	0.471–2.492	0.851	–	–	–
Oral corticosteroid prescribed for exacerbation	1.298	0.466–3.618	0.618	–	–	–

Adjusted for age, gender, smoking status, baseline FEV_1_ (% predicted), COVID-19 vaccination status, ACT score at prior visit 12 months before and GINA step of medication at baseline

*OR* odds ratio, *aOR* adjusted odds ratio, *GINA* global Initiative for asthma

*Statistically significant

### Uncontrolled asthma with ACT score ≤ 15 at enrolment visit

Overall, 31 (14.0%) of them had uncontrolled asthma at enrolment visit with greater proportion observed in those who had COVID-19 than those who did not (19.8% vs. 8.2%). The OR by univariate logistic regression was 2.774 (95% CI = 1.214–6.338, p = 0.016) and the aOR was 5.509 (95% CI = 1.061–28.600, p = 0.042). Specifically, among patients with controlled asthma (ACT score ≥ 16) at the immediate prior visit, 41.4% and 85.3% became uncontrolled (with ACT score ≤ 15) in enrolment visit in the COVID-19 and COVID-19 group respectively. The OR by univariate logistic regression was 8.209 (95% CI = 3.018–22.333, p < 0.001) and the aOR was 7.532 (95% CI = 2.268–25.013, p < 0.001). The results were summarized in Table 2.

### Escalation of asthma maintenance therapy by at least 1 GINA step

Overall, 22 (10.0%) of the patients had escalation of asthma maintenance therapy by at least 1 GINA step with greater proportion observed in those who had COVID-19 than those who did not (15.3% vs. 4.5%). The OR by univariate logistic regression was 3.798 (95% CI = 1.349–10.694, p = 0.012) and the adjusted OR (aOR) was 4.733 (95% CI = 1.151–19.467, p = 0.031). The results were summarized in Table 2.

### ACT score on enrolment visit

**Fig. 1:**
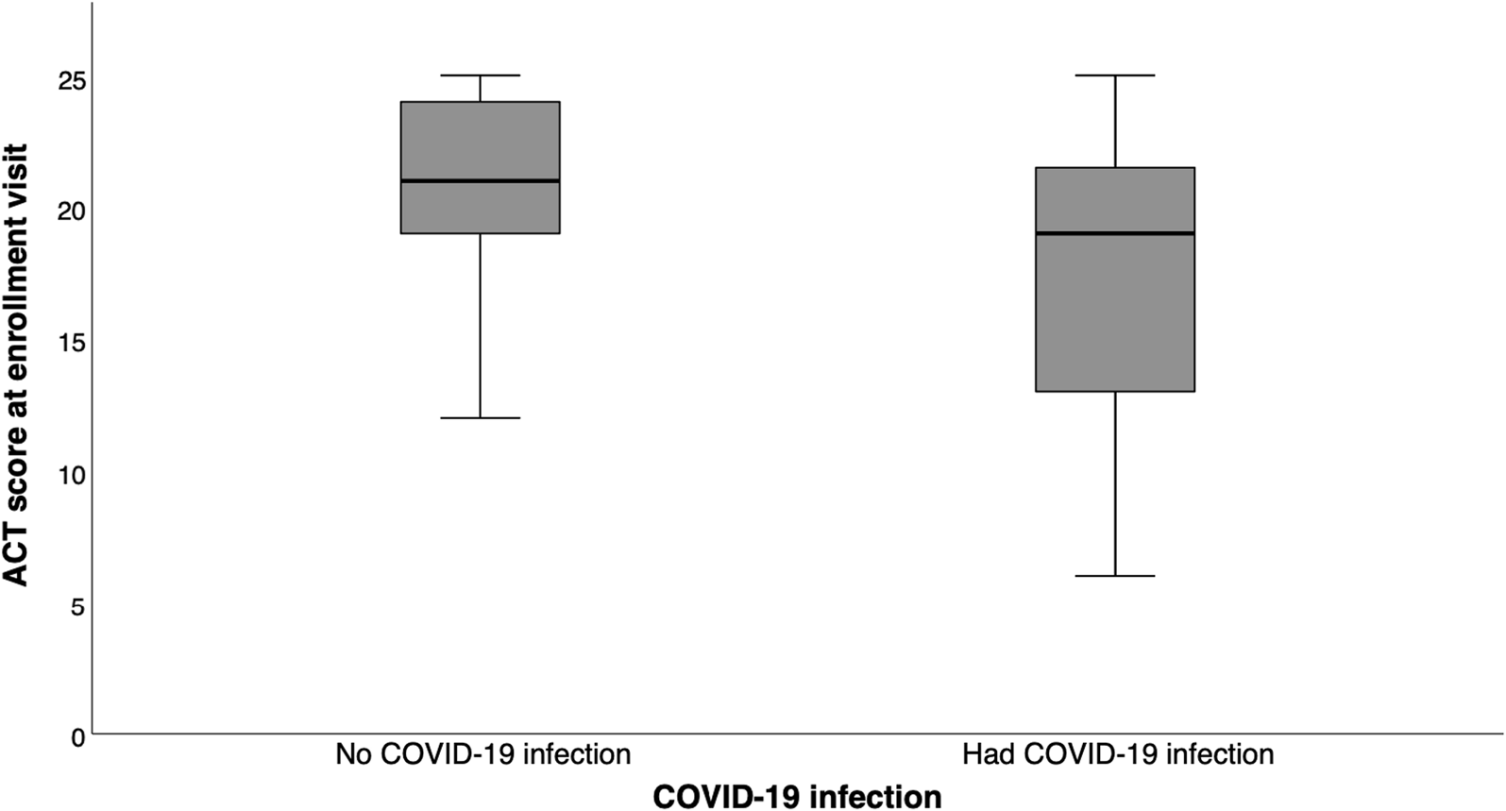
ACT score on enrolment visit among patients with or COVID-19

**Fig. 2:**
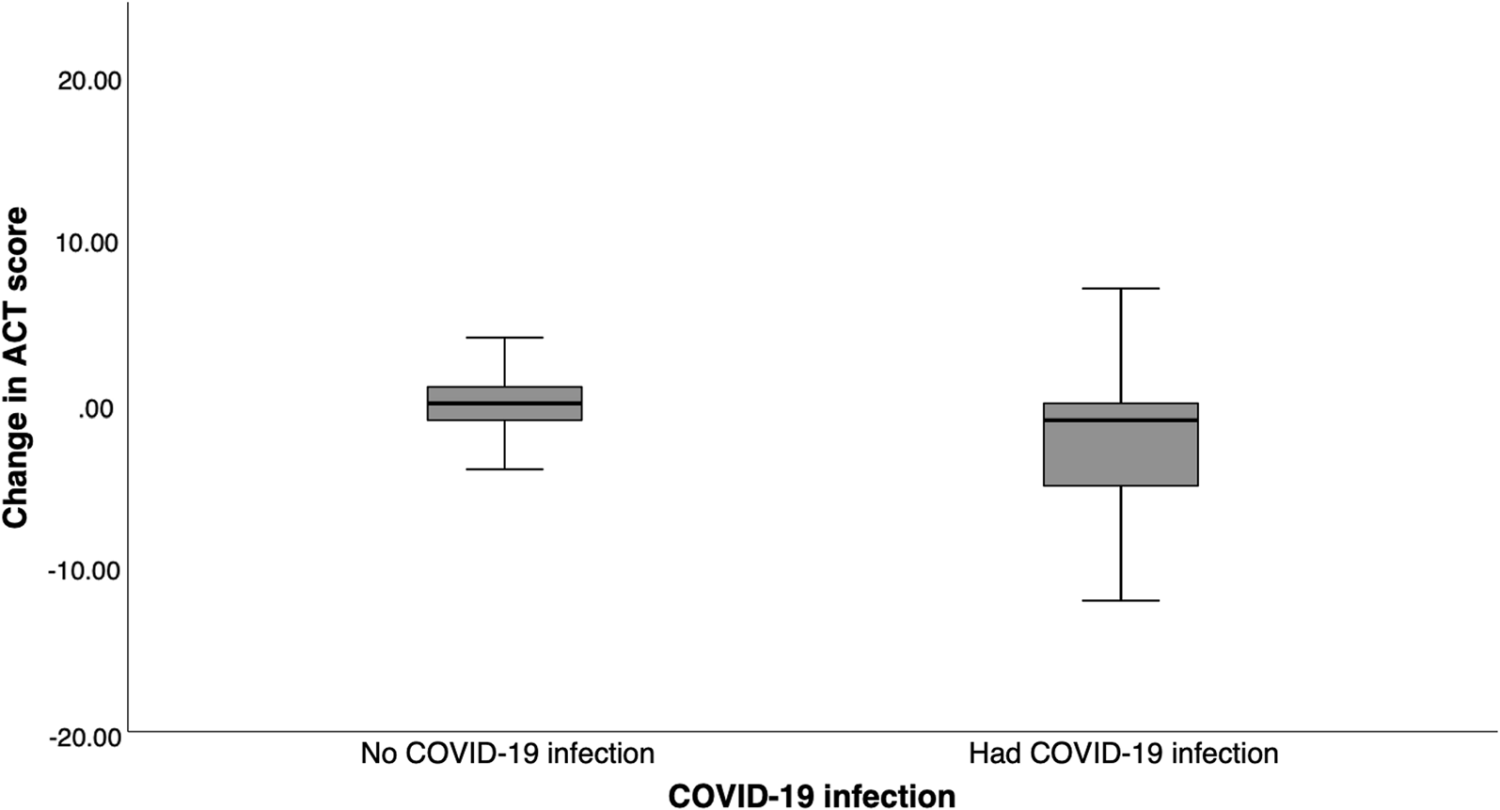
Change in ACT score on enrolment visit among patients with or COVID-19

**Fig. 3:**
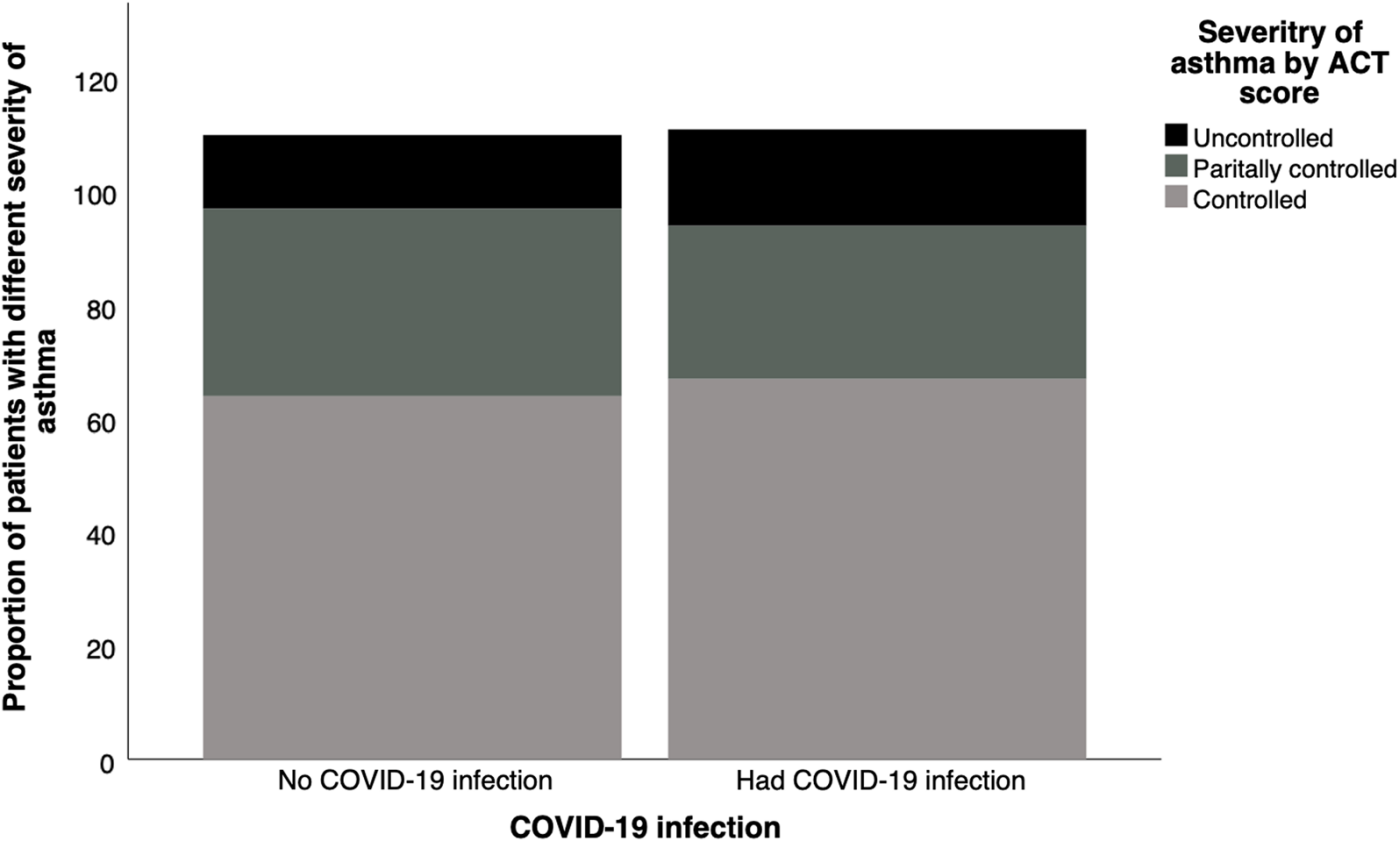
Proportion of patients with controlled, partially controlled and uncontrolled asthma by ACT score on enrolment visit

While the mean ACT was similar between the COVID-19 and non-COVID-19 groups at prior visit 12 months before (20.0 ± 4.5 vs. 20.0 ± 3.5), it was significantly lower in the COVID-19 group on follow-up (17.5 ± 5.3 vs. 20.4 ± 3.9, p < 0.001). The mean reduction in ACT was more marked in the COVID-group (− 2.47 ± 4.96 vs. + 0.40 ± 3.46, p < 0.001). The results were summarized in Figs. 1, 2, 3.

## Discussion

Our study suggested that there was worsening of asthma control after recovery of mild-to-moderate COVID-19. The worsening was consistently reflected in different domains, including a reduction in ACT, an escalation of asthma maintenance therapy and a larger proportion of uncontrolled asthma on follow-up. Our study concurs with the previous findings of worsening of asthma control after COVID-19 which persisted after the recovery from infection. The deterioration is not only subjective as measured by ACT score change, but it is also consequential in the need to escalate the asthma maintenance treatment. [10, 12]. The cases recruited had COVID-19 infection at least 30 days before enrolment. According to local public healthcare policy, those who had completed COVID-19 vaccination only need to have quarantine for 7 days, while those who did not complete vaccination needs 14-days quarantine. All the patients recruited are out of isolation order so that they can visit the clinic for asthma follow up. Most of them also had mild COVID-19 that did not require hospitalization. Given they were all out of the quarantine period for at least 14 days and they had mild COVID-19, our findings can reflect the intermediate effect of COVID-19 on asthma.

Apart from the immediate damage and complications from COVID-19, another area of interest are the persistent consequences from COVID-19. Post-COVID syndrome or long-COVID is a syndrome encompassing a protracted course of various physical and neuropsychiatric symptoms that persist for more than 12 weeks after COVID-19 without an alternative explanation [18–22]. Previous studies did not suggest that premorbid asthma is associated with an increased risk of post-COVID syndrome, but these patients contribute a relatively small subset in most of the published literature [23]. An UK/USA/Sweden cohort of 4182 symptomatic COVID-19 patients found a significantly higher prevalence of pre-existing asthma in COVID-19 patients with persistent symptoms for more than 28 days [24], while a Norwegian study on mixed hospitalized and home isolated COVID-19 cohort identified chronic respiratory disease to be associated with persistent symptoms 6 months after the acute infection [25]. However, unlike the current studies, these results mainly focus on long-COVID with non-specific symptoms rather than asthma-specific symptomatology. Auto-immunity is one of the postulations underlying the associations between asthma and the long-term sequelae after COVID-19 observed in our study [24, 26]. Further research on the underlying pathogenic mechanism of asthma and long-COVID as well as worsening of asthma control are worthwhile, as this might bring about the development of possible preventive measures of long-COVID and worsening of asthma control.

There are a few limitations in our study. First, this study involved only a single centre. However, the respiratory unit in our tertiary medical centre received referrals from all other healthcare sources, and patients diagnosed with asthma were managed in a designated asthma clinic, and the patients in this study have comprehensive clinical data including lung function test results. Some of the data within the study were from retrospective database, including past ACT score 1, asthma medication, comorbidities, spirometry results and COVID-19 vaccination details, with the asthma control assessment repeated at subsequent clinic visit. The most ideal study setting should involve prospective assessment with subsequent follow-up of all the patients. But all the relevant data collected in the study were well-documented within the database and being validated by the authors. The data obtained from the study are deemed very close to a prospective study setting, given the validity of the data within the clinic database. Secondly, the patients were diagnosed to have COVID-19 by RAT or PCR and therefore the measurement of viral load at the time of infection is not possible, but we used the clinical severity as a surrogate marker which should still allow the defining of a well-defined cohort.

## Conclusion

Mild-to-moderate COVID-19 among asthma patients was associated with worsening of asthma symptom, lower ACT score, a greater need for escalation of asthma maintenance therapy and more uncontrolled asthma after recovery.

## Data Availability

The datasets supporting the conclusions of this article are included within the article and no additional data will be provided.

## Abbreviations

ACT: Asthma control test
aOR: Adjusted odds ratio
CI: Confidence interval
CMS: Clinical Management System
COPD: Chronic obstructive pulmonary disease
COVID-19: Coronavirus disease 2019
ePR: Electronic patient record
FEV_1_: Forced expiratory volume in one second
FVC: Forced vital capacity
GINA: Global initiative for asthma
IgE: Immunoglobulin E
IQR: Interquartile range
MID: Minimally important difference
OR: Odds ratio
RAT: Rapid antigen test
RT-PCR: Reverse transcription–polymerase chain reaction
SD: Standard deviation
